# Supplementary material for: Electrically reconfigurable terahertz signal processing devices using liquid metal components
Source: Nat Commun. 2018 Oct 10;9:4202. doi: 10.1038/s41467-018-06463-z (PMC6180054; doi:10.1038/s41467-018-06463-z)
Supplement: Supplementary file 1 — Supplementary Information [file 41467_2018_6463_MOESM1_ESM.pdf]

Electrically Reconfigurable Terahertz Signal Processing Devices using Liquid Metal Components

K. S. Reichel, et al.

Electrically Reconfigurable Terahertz Signal Processing Devices using Liquid Metal Components

Kimberly S. Reichel[1], Nicolas Lozada-Smith[1], IshanD. Joshipura[2], Jianjan Ma[1], Rabi Shrestha[1], Rajind Mendis[1], Michael D. Dickey[2], and Daniel M. Mittleman[1*]

Supplementary Information

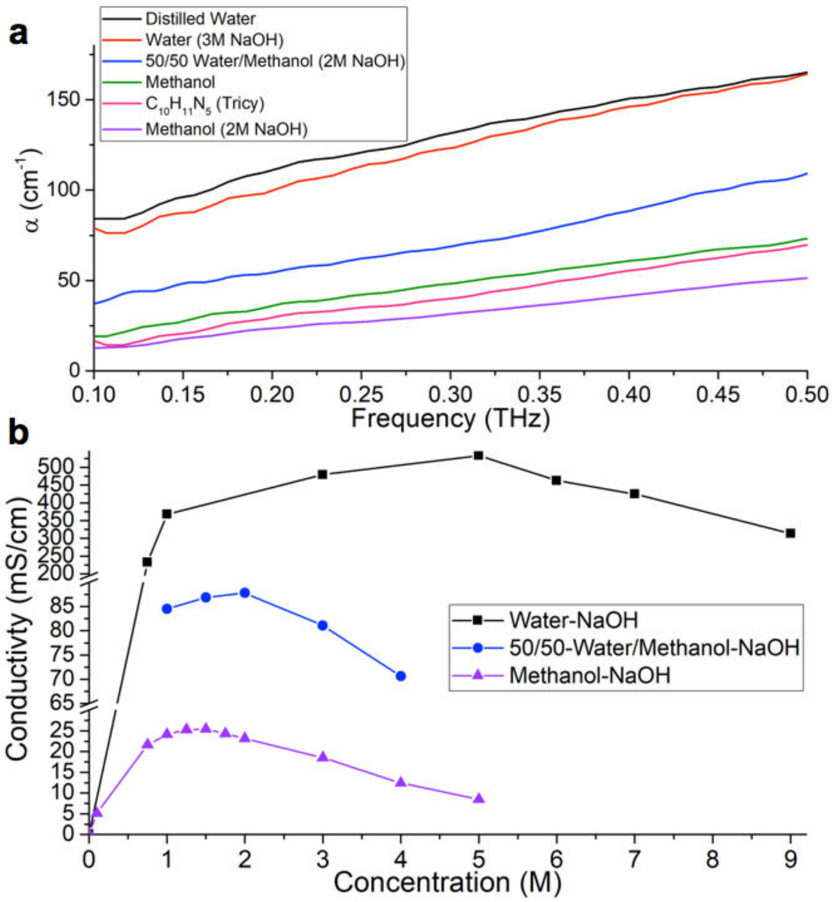

Supplementary Figure 1. Optimization of conductive fluid.

Supplementary Note 1. To operate the liquid metal in this device design, many factors of the surrounding electrolyte must be considered carefully. As candidates for the conductive fluid, we

consider both electrolyte mixtures and ionic liquids, to find an optimized solution which provides adequate DC conductivity and the desired level of THz absorption. In some cases (e.g., the T-junction configuration described in the manuscript), a high absorption coefficient is desired; in others, (e.g., the TE_1 mode add-drop filter, as discussed further below) a low absorption coefficient is necessary. In Supplementary Fig. 1a, we experimentally measured the THz absorption of several candidates using a variable path length liquid transmission cell in a time-domain spectrometer. Since methanol is not as polar as water, the THz absorption for the methanol is lower than that of water. For the electrolyte mixtures, the absorption decreases for increasing concentrations, similar to previous results[1]. In terms of low THz absorption, 2M NaOH (sodium hydroxide) in methanol is the best candidate. We also measure the conductivity of three different mixtures with various concentrations of NaOH. These results are shown in Supplementary Fig. 1b. The maximum conductivity in water (black squares) is 500 mS/cm at concentration of 5 moles/liter. This is an order of magnitude greater than the maximum conductivity for methanol (purple triangles), which is 25 mS/cm at a concentration of 1.5 moles/liter. To balance optimal conductivity and transmission, we consider mixtures of NaOH in water and methanol (blue circles). In general, where THz absorption is not a concern (or when high absorption is desired), we use NaOH in water, but when absorption needs to be minimized, a mixture of NaOH in 50% water and 50% methanol is used.

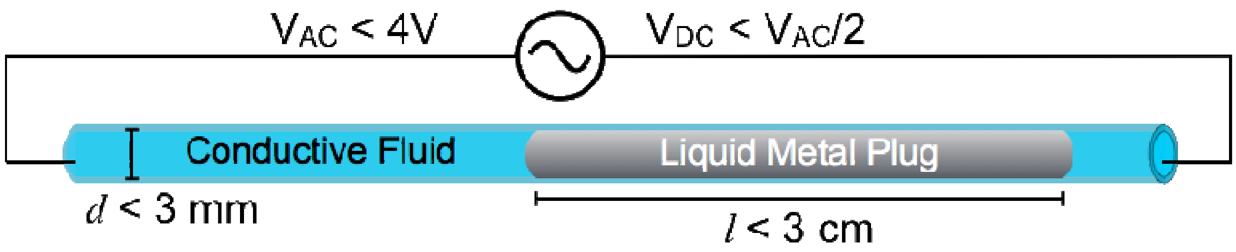

Supplementary Figure 2. Optimization of voltage.

Supplementary Note 2. Oscillatory motion of the plug is achieved when the DC offset is set to zero and therefore the amplitude of AC wave is the defining factor of plug movement in the continuous electrowetting (CEW) manner. It is important to pre-fill the capillary with the electrolyte prior to injecting in the liquid metal to achieve a slip layer such that the metal does not adhere to the glass surface. The optimal liquid metal plug movement includes high velocity and large displacement. In general,optimal displacement is achieved through high voltages, but the voltage must be restricted to avoid plug splitting, pinching, and electrochemical reactions. These limits are described in the diagram.

The ideal operating voltage window depends on the electrolyte and plug length. In principle, the voltage should be less than 1.23 V to avoid hydrogen gas formation, but since there are resistive losses through the electrolyte and the electrolyte-electrode interface, higher values up to 4 V may be used since the applied voltage is not the same as the voltage drop across the length of the metal plug.

The length of the plug (*l)* should stay at or below 3 cm for a 1 mm inner diameter capillary. Beyond this plug length, the plug can start to split when applying an AC voltage. Additionally, a longer plug responds more slowly to an AC voltage. Thus, a shorter plug has the best performance; however, the plug length should be comparable to the THz beam diameter in order to avoid leakage of radiation around the plug ends.

The size of glass capillary inner diameter (*d)* is limited by the capillary length for a given fluid, which is the characteristic length that compares surface tension (force acting up) and gravity (force acting down). If the size of the plug in the vertical direction exceeds the capillary

length, then the effects of gravity will become significant. The corresponding capillary length for gallium alloys is ~ 3 mm (assuming the surface tension to be ~600 mN/m)[2].

Combined, these constraints determine the possible oscillating speeds. In order to move a 8 mm long plug with 8 mm displacement (i.e., completely in and out of the beam path), the AC is limited to low frequencies of ~5 Hz or less. Oscillations as high as 70 Hz have been observed (via high speed camera), but at these frequencies the plug displacement is much smaller (~100s of microns)[3]. In terms of long-term stability, the plug is able to oscillate continuously for days.

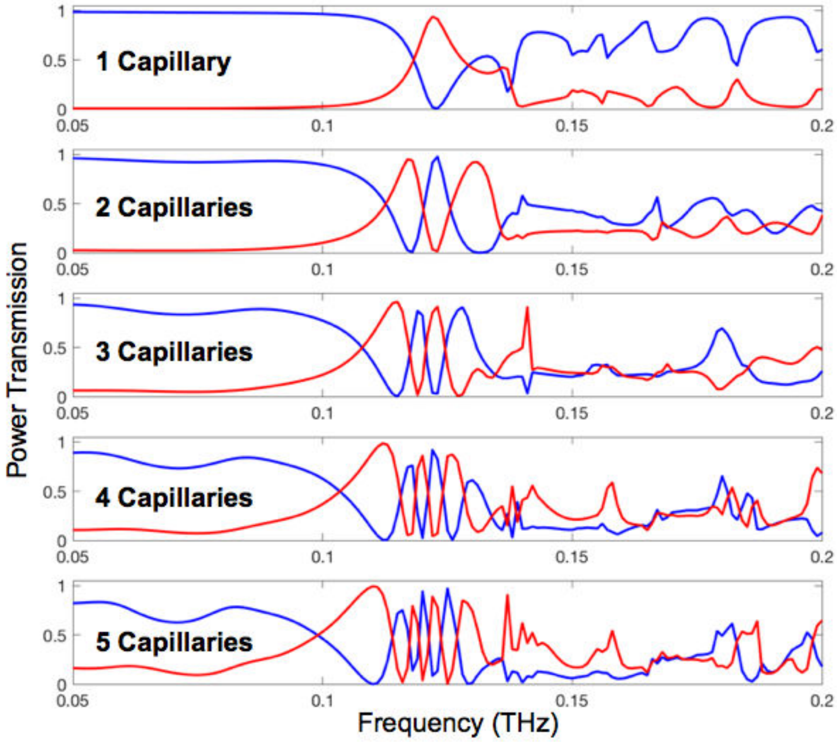

Supplementary Figure 3. 2D FEM simulations for multiple glass capillaries.

Supplementary Note 3. Supplementary Figure 3 shows simulations of multiple glass capillaries in between two parallel PPWGs with 1 mm metal spacing separation. The number of resonant features increases with increasing number of capillaries, and the resonances narrow.

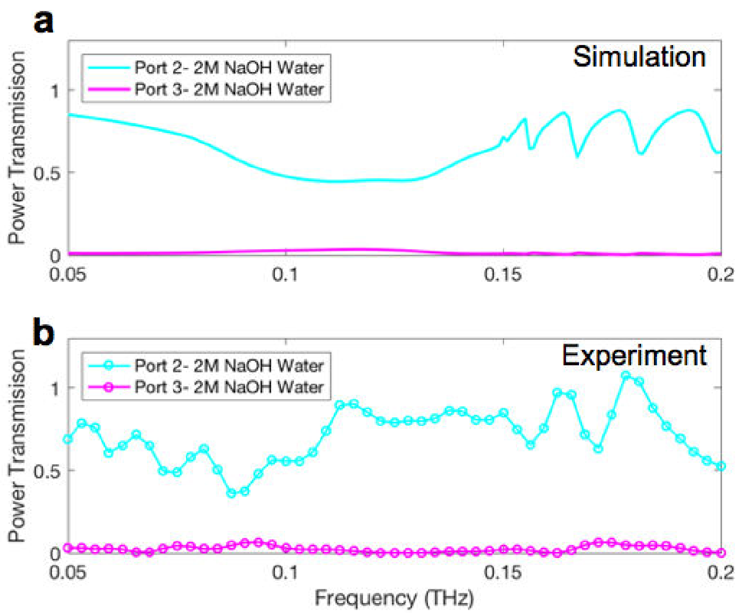

Supplementary Figure 4. Single-capillary between waveguides filled with electrolyte.

Supplementary Note 4. This figure shows FEM simulations and measured results, for the same device as in Fig. 3 of the main text, but in the case when the metal plug is withdrawn and the electrolyte (2M NaOH in water) is in the beam path. We find about 50% transmission through Port 2 in the range 100-150 GHz (cyan) and minimal transmission through Port 3 (magenta).

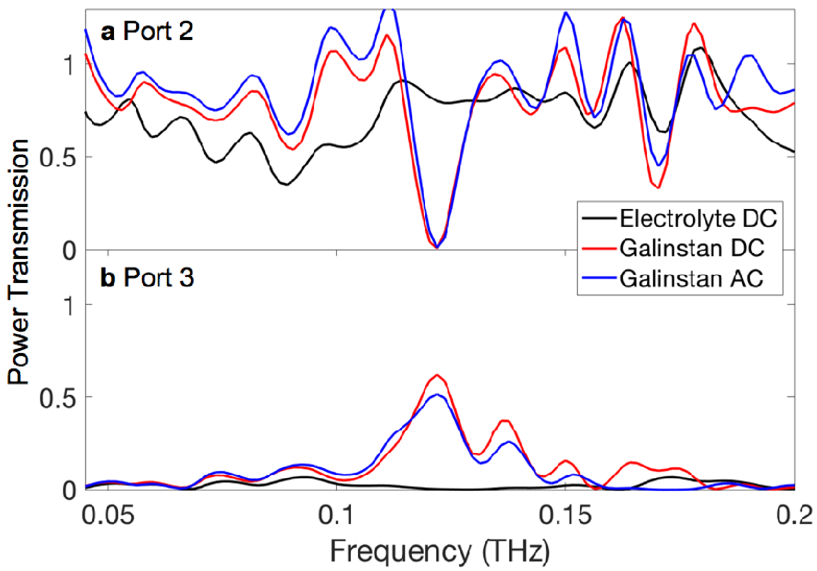

Supplementary Figure 5. Single-capillary plug with AC oscillation.

Supplementary Note 5. When the DC offset is set to zero, the amplitude of the AC wave is the defining factor of plug movement in the continuous electrowetting (CEW) method.The capillary is filled with 1.5 M NaOH in water and plug of 9 mm length. Due to the factors described above, the frequency is kept at 2 Hz to avoid splitting of the liquid metal plug, and the voltage is kept at 2 $V_{p\text{-}p}$ to avoid electrochemical reactions. These conditions cause the plug to oscillate with a displacement of ~7 mm. We obtain the power transmission at the output of Port 2 and Port 3, as shown in panels **a** and**b** of Supplementary Fig. 5, respectively. We trigger the data acquisition to when the Galinstan plug is in the beam path; this is the case when the resonance is present.The AC measurements (blue curves) are compared to the static DC electrolyte (black curves) and static DC Galinstan (red curves)measurements. These data demonstrate that, for an AC oscillation of 2 Hz, the electromagnetic performance of the device (blue curves) is very similar to the performance measured in a static configuration with DC voltages (red curves).

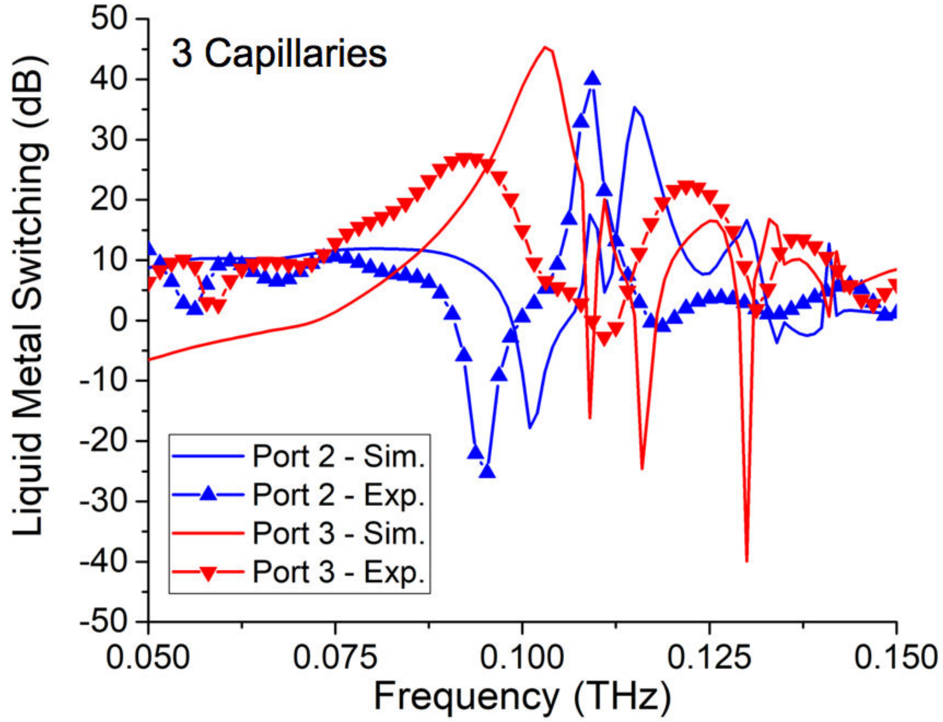

Supplementary Figure 6. Switching ratios for three-capillaries with 1.2 mm outer diameter.

Supplementary Note 6. The result shown in Supplementary Figure 6 is similar to Fig. 4 of the main text but here for the larger capillary device used for the modulated data stream transmission. The resonant frequency of the experimental device at 90 GHz (symbol lines) is shifted to a slightly lower frequency than predicted by simulation at 100 GHz (solid lines). The switching ratio between the 000 and 101 configurations at the experimental resonance is +30 dB for Port 3 (red) and –30 dB for Port 2 (blue). Since thedata stream measurements are fixed at a carrier frequency of 98 GHz, the results in Fig. 6 of the main text are on the side of the resonant peak yielding a lower switching ratio than what is observed in static measurements at the resonant frequency.

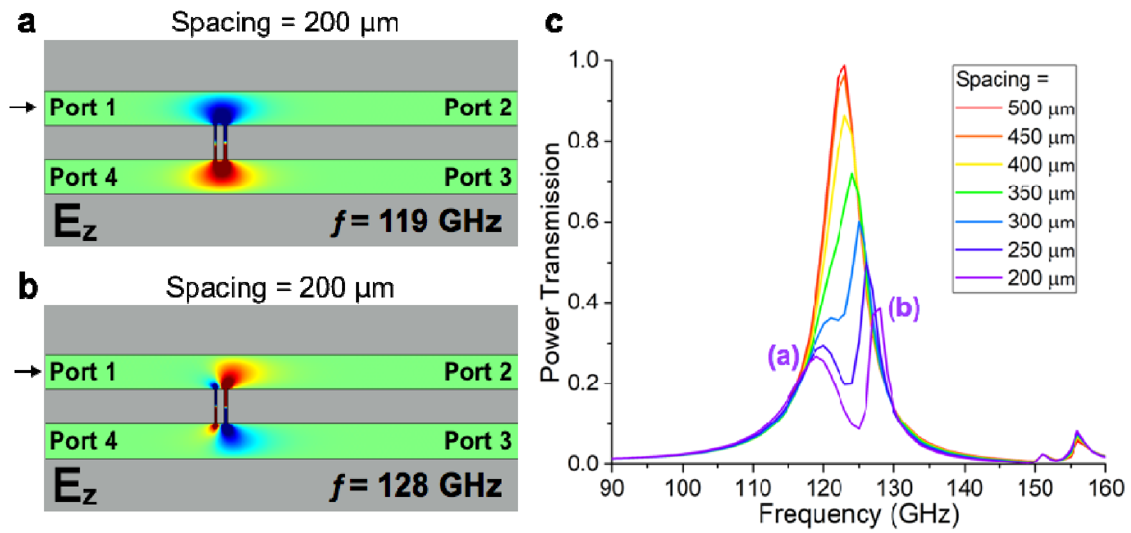

Supplementary Figure 7. FEM simulation of simplified coupling geometry between two waveguides.

Supplementary Note 7. To explain the emergence of the narrow resonant features, we simplify the problem of a 0.1 mm thick glass capillary with 0.8 mm inner diameter (i.e., the situation shown in Fig. 2 of the main text) to that of two 0.1 mm wide rectangular gaps of air with 0.8 mm spacing inbetween the two waveguides. Ordinarily, when there is a single rectangular gap of 0.1 mm width and 1 mm thickness, 25% of the light would couple out of each port. However, with a second rectangular gap of the same width and spaced 0.8 mm apart, the resonances of the two gaps couple, allowing for nearly 100% transmission through Port 3. Panels **a,b** of Supplementary Fig. 7 show the E_z component (in the direction of propagation), showing the excitation of the gaps at the resonance. Panel **c** shows the power transmission through Port 3 for various gap spacings. For a narrow spacing of 200μm, we clearly see two resonant features: **a,** one symmetric and **b,** one anti-symmetric. As the gap widens, these resonances couple together and the total power transmission through Port 3 increases. A maximum is reached at 500 μm, at a gap spacing of 600 μm, which closely corresponds to the λ/4 condition of the resonance at 123 GHz.

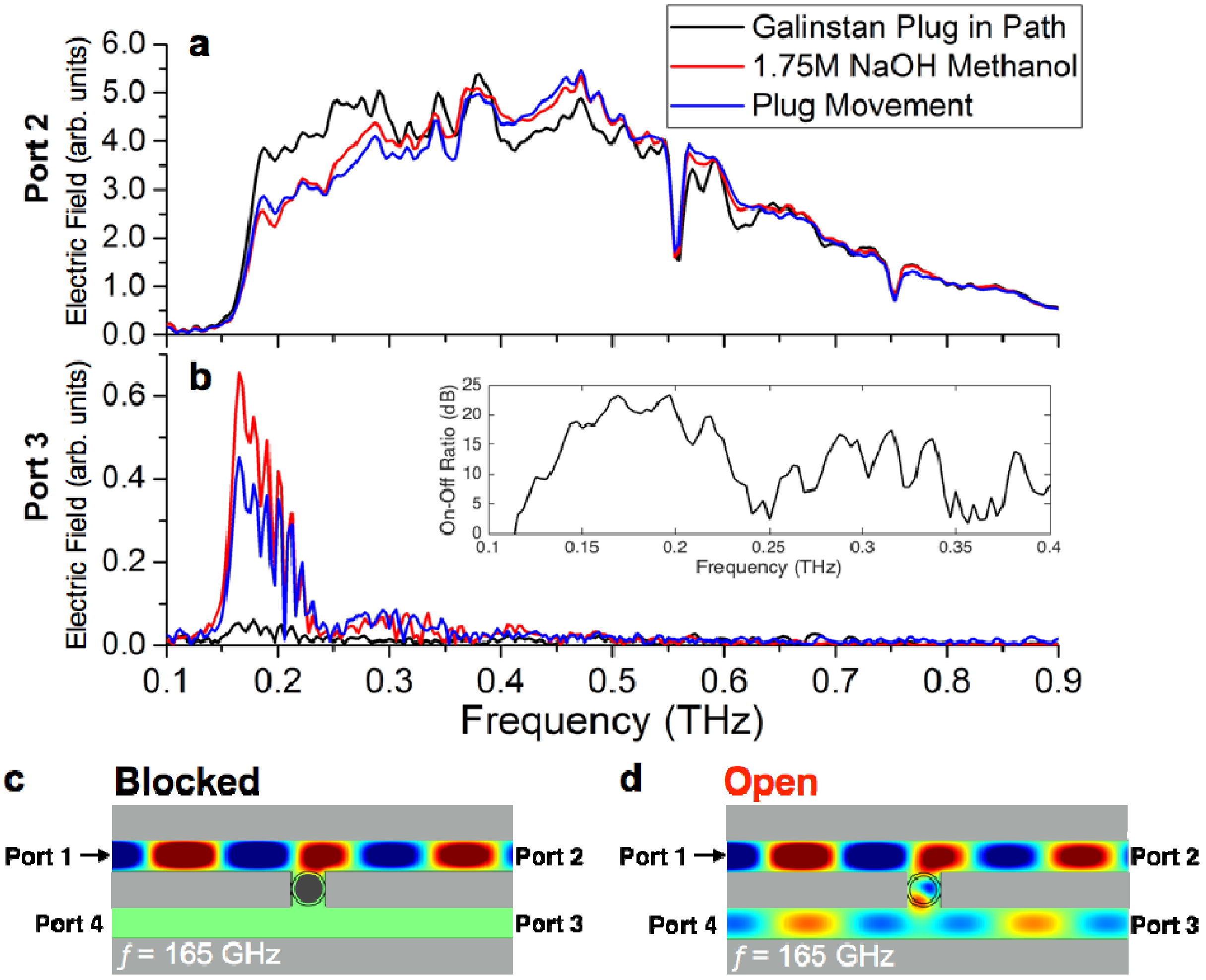

Supplementary Figure 8. Add-drop filter with TE_1 mode excitation.

Supplementary Note 8. All of the above measurements have employed the TEM mode of the parallel-plate waveguides; however, interesting behavior is also observed with the TE_1 mode is used instead. In Supplementary Figure 8, we show the transmission through Port 2 (panel **a**) and through Port 3 (panel **b**). In **b**, only a small fraction of the light is coupled to the bottom waveguide. The capillary is filled with a low-absorbing conductive fluid (1.75 M NaOH in methanol) to move a 5 mm long plug of liquid metal Galinstan. Using a DC bias, the Galinstan plug is moved into the beam path (black curves) and out (red curves). AC measurements (blue

curves; square wave voltage at 2 Hz and 4 V peak-to-peak) give similar results to the static DC measurements (red curves). In these measurements, the plug moves only about 3 mm, which is smaller than the size of the THz beam, explaining the small difference between the red and blue curves in **b**. The inset shows an on-off ratio between Port 2 and Port 3 of more than 20 dBat the design frequency (165 GHz). In this figure, we also show the electric field extracted from FEM simulations; panel**c** shows the closed configuration (metal plug in) and panel**d** shows the open configuration (metal plug out). Note that in the TE_1 mode case, the coupling between the two waveguides involves significant transmission of radiation through the electrolyte-filled capillary, not merely through its side walls. This is in contrast to the TEM case (Fig. 2 of the main text), where only the capillary side walls are involved, which relaxes the requirement for low THz absorption in the liquid electrolyte.

Supplementary References

[1] P.U. Jepsen and H. Merbold, J. Infrared, Millimeter, Terahertz Waves **31**, 430-440 (2010).

[2] M.R. Khan, C.B. Eaker, E.F. Bowden, and M.D. Dickey, Proc. Natl. Acad. Sci. **111**, 14047-14051 (2014).

[3] M.R. Khan, C. Trlica, J.-H. So, M. Valeri, and M.D. Dickey, ACS Appl. Mater. Interfaces **6**, 22467-22473 (2014).
